# PD‐1/PD‐L1 co‐location: A novel biomarker for immunotherapy response in non‐small cell lung cancer

**DOI:** 10.1111/1759-7714.14436

**Published:** 2022-04-28

**Authors:** Xiayao Diao, Chao Guo, Shanqing Li

**Affiliations:** ^1^ Department of Thoracic Surgery Peking Union Medical College Hospital, Chinese Academy of Medical Sciences and Peking Union Medical College Beijing P. R. China

According to the Global Cancer Statistics 2020, primary lung cancer is the deadliest cancer worldwide.^1^ Compared with other countries in the world, it is significantly more observable in China.^2^, ^3^ Non‐small cell lung cancer (NSCLC) is the most common type of lung cancer. In the past decades, the treatment of NSCLC has changed drastically owing to the advent of immunotherapy. The programmed cell death protein 1 (PD‐1)/programmed death‐ligand 1 (PD‐L1) axis has become the most important therapeutic target for the treatment of locally advanced/metastatic NSCLC.^4^, ^5^ Pembrolizumab is the first PD‐1 inhibitor to receive U.S. Food and Drug Administration approval for the treatment of locally advanced/metastatic NSCLC patients with a PD‐L1 tumor proportion score ≥ 50% as assessed by the Dako PD‐L1 immunohistochemistry 22C3 pharmDx assay. At present, detection of PD‐L1 expression by immunohistochemistry is the ultimate companion test to guide treatment decisions in clinical practice; however, the heterogeneity of PD‐L1, the limited response rates even among patients with high PD‐L1 expression, and the response records in patients with low PD‐L1 expression suggest that PD‐L1 detected by immunohistochemistry is not a perfect biomarker.^6^ Indeed, irrespective of PD‐L1 expression detected by immunohistochemistry, regimens of immunotherapy combined with chemotherapy have been reported to show high objective response rates.^7^

In recent years, several studies have explored the relationship between PD‐1/PD‐L1 spatial and immunotherapy response. For example, by simultaneously detecting PD‐1 and PD‐L1 signals in serial tissue sections, followed by quantification via spatial analysis algorithms, Giraldo et al.^8^ found that the proximity of PD‐1^+^ to PD‐L1^+^ cells in Merkel cell carcinoma was associated with the immunotherapy response. In addition, multiplex immunohistochemistry technique is also a convincing method to detect PD‐1/PD‐L1 interaction.^9^ By using this technique to detect the localization of PD‐1 and PD‐L1, Johnson et al.^10^ also found that the co‐location scores of PD‐1 and PD‐L1 were associated with the immunotherapy response in advanced melanoma patients. However, compared with traditional PD‐L1 expression assays, it remains unknown whether PD‐1/PD‐L1 co‐location scores can more accurately identify patient candidates for treatment with immunotherapy in NSCLC.

In a study recently published in *Clinical Cancer Research*, titled “Association of PD‐1/PD‐L1 Co‐location with Immunotherapy Outcomes in Non‐Small Cell Lung Cancer,” Gavrielatou et al.^11^ assessed the co‐location of PD‐1 and PD‐L1 on a retrospective cohort of 154 advanced NSCLC patients treated with immune checkpoint inhibitor. Expression of PD‐1 and PD‐L1 was detected by multiplex quantitative immunofluorescence. The co‐location of PD‐1 and PD‐L1 were defined as expression in the same pixels at 0.5 μm resolution and the co‐location scores were measured through an algorithm to define overlapping areas. The authors found that the co‐location score of PD‐1 and PD‐L1 was significantly associated with best overall response (BOR; *p* = 0.001), longer progression‐free survival (PFS; *p* = 0.034, hazard ratio [HR] = 0.64, 95% confidence interval [CI]: 0.43–0.97), and better overall survival (OS; *p* = 0.025, HR = 0.60, 95% CI: 0.39–0.93) after treatment with immune checkpoint inhibitors in the second/subsequent line, while PD‐L1 tumor proportion score measured by immunohistochemistry was associated with better PFS (*p* = 0.030, HR = 0.47, 95% CI: 0.29–0.89). However, PD‐L1 of tumor compartment measured by quantitative immunofluorescence was not associated with either BOR or OS. Also, colocation score of PD‐1 and PD‐L1 did not show any significant association with PD‐L1 expression measured by either immunohistochemistry or quantitative immunofluorescence.

PD‐L1 has long been the only molecular target of immunohistochemistry companion diagnostic tests. Since the main effect of PD‐1/PD‐L1 axis drugs is to inhibit the interaction of PD‐1 and PD‐L1, this study attempted to use the parameters of the interaction between PD‐1 and PD‐L1 as the targets of the companion diagnostic test. It indicated that quantitative immunofluorescence could reflect the interaction between PD‐1 and PD‐L1 and thereby accurately mark the active target of immune checkpoint inhibitors in NSCLC. There was no observable correlation between the co‐location score and the expression of PD‐L1, but the co‐location score could effectively sense the actual target of the immunotherapy. Moreover, co‐location score significantly correlated with the prognosis of advanced/metastatic NSCLC patients received immunotherapy and is a potential biomarker for predicting immunotherapy outcomes.

PD‐1 is an important immunosuppressive molecule under physiological conditions. It prevents autoimmune diseases caused by immune cell overactivation through downregulating the response of immune system to normal cells. PD‐1 activates downstream pathways, such as ERK, PI3K‐AKT, and RAS pathways, by directly binding to PD‐L1/PD‐L2, and thereby reducing cell activity and lead to cell exhaustion.^12^ In malignant tumors, PD‐L1 is mainly expressed by tumor cells, so the above mentioned physiologically protective PD‐1/PD‐L1 interaction mechanism has become the main mechanism of tumor immune evasion.^13^ Therefore, blocking the PD‐1/PD‐L1 interaction has become the main goal of tumor immunotherapy.^14^ In other words, the efficacy of tumor immunotherapy depends on the interaction of PD‐1 and PD‐L1, rather than the individual expression of PD‐1 or PD‐L1. Indeed, PD‐L1 has not shown predictive value in the immunotherapy of NSCLC. However, as immunotherapy has shown better efficacy as a second‐line or subsequent line of treatment in NSCLC compared with chemotherapy, it has been commonly used without an approved companion diagnostic test at the cost of a response rate limited to 14%–20% and 40% of patients will have disease progression.^7^, ^15^ This study is the first to explore the predictive value of the PD‐1/PD‐L1 co‐location score in the response to immunotherapy in NSCLC and found that the co‐location score was significantly associated with improved immunotherapy outcomes for second‐line or subsequent line treatment cases. The results of this study suggest that receptor/ligand proximity is an essential biological process in tumor microenvironment, whereas high receptor or ligand expression is not.

This study has several limitations. First, it was performed on a retrospectively collected cohort with limited case number and heterogeneity in the treatment of enrolled patients. Second, this study used tissue microarrays with limited tissue sample size, rather than traditional tissue sections. Third, most of the tissues analyzed in this study were from the primary tumor, thus the results could not reflect the PD‐L1 expression status of metastatic tissues. Finally, this study did not enroll NSCLC patients without immunotherapy, therefore it cannot further demonstrate whether the PD‐1/PD‐L1 co‐location score can be used as a prognostic biomarker.

Despite the limitations mentioned above, this study by Gavrielatou et al. demonstrated that co‐location score could accurately depict the interaction between PD‐1 and PD‐L1, and leading to more accurate identification of candidates for immunotherapy when compared to PD‐L1 immunohistochemistry.

## CONFLICT OF INTEREST

The author declares no competing interests.

## CONSENT FOR PUBLICATION

Not applicable.

## Data Availability

Not applicable.
